# Comparison of Perceived Weight Discrimination between Polish and German Patients Underwent Bariatric Surgery or Endoscopic Method versus Conservative Treatment for Morbid Obesity: An International Multicenter Study

**DOI:** 10.3390/nu14132775

**Published:** 2022-07-05

**Authors:** Karolina Hoffmann, Anna Paczkowska, Wiesław Bryl, Kinga Marzec, Jonas Raakow, Matthias Pross, Rafael Berghaus, Elżbieta Nowakowska, Krzysztof Kus, Michał Michalak

**Affiliations:** 1 Department of Internal Diseases, Metabolic Disorders and Arterial Hypertension, Poznan University of Medical Sciences, 60-806 Poznań, Poland; wieslawbryl@ump.edu.pl; 2 Department of Pharmacoeconomics and Social Pharmacy, Poznan University of Medical Sciences, 60-806 Poznań, Poland; aniapaczkowska@ump.edu.pl (A.P.); kinga.marzec@onet.pl (K.M.); kkus@ump.edu.pl (K.K.); 3 Department of Surgery, Campus Charité Mitte, Campus Virchow Klinikum, Charité—Universitätsmedizin Berlin, 10117 Berlin, Germany; jonas.raakow@charite.de; 4 Department of Surgery, DRK Kliniken Berlin, Köpenick, 12559 Berlin, Germany; m.pross@drk-kliniken-berlin.de (M.P.); r.berghaus@drk-kliniken-berlin.de (R.B.); 5 Department of Pharmacology and Toxicology, Institute of Health Sciences, Collegium Medicum, University of Zielona Góra, 65-516 Zielona Góra, Poland; elapharm@ump.edu.pl; 6 Department of Computer Science and Statistics, Poznan University of Medical Sciences, 60-806 Poznań, Poland; michal@ump.edu.pl

**Keywords:** obesity, morbid obesity, discrimination, weight stigma, obesity stigma, bariatric surgery

## Abstract

**Objectives:** The aim of this study was to compare the level of discrimination among patients with obesity living in Poland and Germany. **Methods:** This was a retrospective cross-sectional international multicenter survey study including 564 adult participants treated for morbid obesity at selected healthcare facilities in Germany (210 patients) and in Poland (354 patients). Discrimination was evaluated using a custom-made questionnaire based on the related literature. **Results:** The level of obesity discrimination did not differ between German and Polish patients (*p* = 0.4282). The presence of obesity was reported to be associated to a large or a very large extent with the feeling of social exclusion and discrimination by 46.63% of German participants and 42.09% of Polish ones (*p* = 0.2934). The mean level of discrimination related to the lack of employment was higher in patients who underwent bariatric surgery or endoscopic method than in those who underwent conservative treatment (for Germany: 2.85 ± 1.31 (median, 3) vs. 2.08 ± 1.31 (median, 1), *p* = 0.002; for Poland: 2.43 ± 1.15 (median, 2) vs. 1.93 ± 1.15 (median, 1), *p* = 0.005). The level of discrimination was associated with sex, age, the degree of obesity, and treatment-related weight loss (*p* < 0.05). **Conclusions:** Our findings confirm that obesity significantly affects the social and economic well-being of patients. There is a great need to reduce weight stigma and to take measures to alleviate the socioeconomic and psychological burden of obesity.

## 1. Introduction

Obesity and its health consequences are an emerging public health issue. The prevalence of obesity has been increasing worldwide. According to current data, more than one-fifth of Europeans are obese, including 21.5% of men and 24.5% of women. These rates are threefold higher than those reported for 1986, when the European Association for the Study of Obesity was established to address the rising problem of obesity [1,2]. Data from the cross-sectional observational NATPOL study, which assessed the prevalence of cardiovascular risk factors in Poland, revealed that 50% of Polish adults aged 18–79 years are overweight, while obesity was reported for 22% [3]. In Germany, the prevalence of overweight and obesity among primary care patients is estimated at 60% and 20%, respectively [4]. The World Health Organization recognized obesity as a major risk factor implicated in the development of noncommunicable diseases such as diabetes, hyperlipidemia, arterial hypertension, cardiovascular disease, and cancer [5]. Moreover, owing to its close association with type 2 diabetes, the term “diabesity” was coined to reflect the combination of these two entities [6]. The increasing prevalence of obesity is also paralleled by a dramatic rise in the rates of obesity-related disabilities as well as by a reduced quality of life [7].

In the health assessment of patients with morbid obesity, the physical, psychological, social, and economic aspects of life should be considered. The physical condition of these patients has been addressed already in early research on obesity. Later, investigators focused also on the other aspects. In the 1985 consensus statement of the National Institute of Health, it was acknowledged that obesity has enormous psychological consequences [8]. Subsequent research showed a poorer quality of life, body image disturbances, lower self-esteem, and concomitant psychiatric disorders among patients with obesity [9,10,11,12]. The strongest negative effects of excessive body mass are observed among individuals with morbid obesity. It was reported that patients awaiting bariatric surgery would even prefer to have a serious handicap (e.g., deafness, blindness, or a history of leg amputation) rather than to stay morbidly obese, and the majority of them would choose to be a normal-weight individual over a morbidly obese multimillionaire [10]. Weight stigma is thought to be one of the possible mechanisms that account for the worsening of the psychological status among people with obesity [10,12]. Prejudice against obesity is commonly reported worldwide and is the source of complaints about discrimination voiced by affected individuals [13,14]. Weight stigma and discrimination were shown to adversely affect mental health outcomes, including well-being [15], depression [16,17], self-esteem and self-acceptance [16,18], and body image dissatisfaction [16,19]. 

A study conducted by Geller et al. revealed that preoperative assessment of body-related emotional distress patterns among bariatric surgery candidates may enable professionals to identify potential postoperative risks of suicide, anxiety, and decreased weight loss. The relationship between the body-related emotional distress cluster and outcome measures is culture-dependent [20]. The most economically developed countries, such as the US or Germany, present a more individualistic culture and may thus provide less social support, which reduces patients’ ability to cope with the drastic changes associated with bariatric surgery [21]. Recent studies have proved that high preoperative levels of body image dissatisfaction are known to be associated with weight-related stigma and overt discrimination [22].

Effective treatment of morbid obesity should include interventions aimed at improving not only the physical but also mental, social, and economic status of the patient. The management of obesity differs significantly between the European Union countries. In a comparison of the German and Polish strategies, Germany clearly outperformed Poland in terms of financing therapeutic educational programs for obesity. However, there are limited literature data on the socioeconomic status of patients with morbid obesity as well as the levels of perceived discrimination in European Union countries. Moreover, only a few studies assessed the association between perceived discrimination and different psychological, social, and economic measures [23,24]. Cultural beliefs and attitudes were identified as significant factors that contribute to the development of eating disorders and weight stigma [23]. A systematic international comparison of weight stigma revealed that there were more similarities than differences in the nature, frequency, and interpersonal sources of weight stigma experienced by people across six countries (Australia, Canada, France, Germany, the United Kingdom, and the United States) [24]. Nevertheless, German participants reported a higher frequency of weight stigma across their lifespan, but a lower distress associated with stigmatizing experiences than participants in the remaining five countries. 

The aim of the present study was to compare the level of perceived discrimination between patients treated for morbid obesity in Poland and Germany. Moreover, we assessed associations between the level of discrimination and different demographic and clinical parameters such as sex, age, duration of diabetes, or the type of treatment.

## 2. Materials and Methods

### 2.1. Study Population

A retrospective cross-sectional international multicenter survey design was adopted for this study. Based on the inclusion and exclusion criteria described below, we enrolled 564 adult participants (210 German and 354 Polish patients) treated for morbid obesity between January 2018 and December 2019 at selected healthcare facilities in Germany and in Poland. The inclusion criteria were as follows: body mass index—BMI > 40 kg/m^2^ in the medical history before the implementation of bariatric/endoscopic or conservative treatment of obesity, presence of obesity-related comorbidities, bariatric/endoscopic or conservative treatment for obesity during the study period, age 18 years or older, and the ability to understand and comply with the study procedures. The decision to include a patient in the study was made by the attending physician based on the adopted inclusion and exclusion criteria. Prior to enrolment, each eligible participant provided written informed consent to participate in the study.

### 2.2. Study Technique

The level of perceived discrimination was evaluated using a validated custom-made questionnaire based on the related literature [25]. The level of discrimination in the study group was assessed in the time horizon of the last 6 months. The questionnaire consisted of 20 closed questions regarding weight discrimination, disease duration, current BMI, type of bariatric surgery or endoscopic method, time since bariatric surgery or endoscopic method, types of intervention and application areas as part of the conservative treatment of morbid obesity, and duration of comorbidities if present. There were five questions assessing the level of discrimination due to obesity. In particular, they concerned employment problems, loss of income, a sense of social exclusion or discrimination, a sense of shame associated with obesity, and difficulties in establishing relationships. For all the questions, a 5-point answer scale was used (scoring range 1–5). The level of perceived discrimination ranged from 5 to 25 points, where 25 indicated the highest level of perceived discrimination by the patient, and 5—the lowest level of discrimination. The study questionnaire was accepted by national consultants in the field of obesity. To evaluate the psychometric properties of the German and Polish version of the questionnaire, it was pretested on a sample of 150 patients with obesity (a representative sample including 75 patients from Poland and 75 patients from Germany). Subsequently, it was possible to revise the questions if needed. The results of the pretest were included in the final study, because the pretest did not yield any major modifications of the instrument. The internal consistency of the weight stigma questionnaire is the extent to which the items are correlated. It was determined by calculating the Cronbach’s α. A good internal consistency (0.7 ≤ α ≤ 0.9) can be seen as a precondition of the fact that the summation of the single item scores to a total score is meaningful. The Cronbach’s α was 0.84 and 0.86 for the total score among German and Polish patients, respectively. 

Each patient responded to the questionnaire independently. Complete questionnaires (100% filled in by the patients) were included in the survey. All the information obtained during the study was fully confidential because patient data were anonymized. Therefore, the research project did not violate the Personal Data Protection Act. The study protocol was approved by the ethics committees of the Poznan University of Medical Sciences and the Charité–Universitätsmedizin Berlin (no. KB 326/19; Poznan University of Medical Sciences Bioethics Committee; issued on 7 March 2019). 

### 2.3. Statistical Analysis

Quantitative parameters were presented using mean, median, and standard deviation. Categorical data were presented as counts and percentages. Results obtained for individual study groups were compared using the Student’s t-test. Data that did not follow the normal distribution in the Shapiro–Wilk test were compared using the Mann–Whitney test. The chi-square test for independence was used to analyze categorical data. The relationship between the level of discrimination and study parameters was analyzed using a multiple regression analysis. For categorical data, the coefficient for a specified level was compared to the reference level.

The analysis was performed using the statistical package Statistica v. 13.3 (TIBCO Software Inc. (2017). Statistica (data analysis software system), version 13. http://statistica.io, accessed on 15 May 2022. All results were considered significant for *p* < 0.05.

## 3. Results

### 3.1. Characteristics of the Study Group

Based on the adopted inclusion criteria, we selected a target group of 564 adult patients (210 German patients and 354 Polish patients), who underwent treatment for morbid obesity. German and Polish patients did not differ in terms of sex, age, current BMI, current degree of obesity, duration of the disease, level of education, and type of treatment (*p* > 0.05). The sociodemographic characteristics of the sample are shown in Table 1. 

Over one-fourth of patients in each group (27.48%) underwent bariatric surgery or endoscopic method (73 German patients and 82 Polish patients), and less than three-quarters (72.52%) were subject to a conservative weight reduction treatment (137 German patients and 272 Polish patients). The most common comorbidities in both groups were hypertension (47.61% and 45.48% of German and Polish patients, respectively), type 2 diabetes mellitus (28.57% and 12.71%), coronary heart disease (24.76% and 24.85%), and dyslipidemia (19.04% and 17.79%).

### 3.2. Assessment of the Level of Discrimination

The analysis based on the Mann–Whitney U test revealed that the general level of perceived discrimination due to obesity did not differ between German and Polish participants (*p* = 0.4282) (Table 2). However, significant differences were noted between both groups in the context of a loss of income (*p* = 0.0018); the level of perceived discrimination was significantly higher among Polish group (1.96 ± 1.11 vs. 1.59 ± 1.00). In the German group, 22.86% of patients reported that obesity was the reason for the lack of employment (to a very high or high degree), as compared with 10.73% of patients from Poland (*p* < 0.0001). Obesity as the cause of reduced income (to a very high or high degree) was reported by 7.14% and 9.03% of participants from Germany and Poland, respectively (*p* = 0.4322). The feeling of social exclusion and discrimination (to a very high or high degree) in association with obesity was reported by 46.63% and 42.09% of German and Polish participants, respectively (*p* = 0.2934). A high degree of shame related to obesity was reported more often by Polish than by German participants (87.85% vs. 77.14%, *p* = 0.0008). Finally, a high or very high degree of difficulty in establishing relationships was reported by 19.52% and 17.51% of German and Polish patients (*p* = 0.5503).

### 3.3. Assessment of the Level of Discrimination Depending on the Type of Treatment

The study showed that the general level of perceived discrimination was similar between patients who underwent bariatric surgery or endoscopic method and those underwent conservative treatment in both in the German (14.19 ± 4.88 (B) vs. 12.64 ± 4.53; *p* = 0.066) and Polish groups (14.50 ± 3.84 (B) vs. 13.32 ± 3.75; *p* = 0.0562)) (Table 2). However, significant differences depending on the type of treatment were noted in both groups in the context of employment problems. The level of perceived discrimination in the above subscale among patients from Poland and also from Germany was significantly higher among bariatric or endoscopic treated patients compared with conservatively treated patients (Poland: 2.43 ± 1.15 (B) vs. 1.93 ± 1.15 (C); *p* = 0.0028; Germany: 2.85 ± 1.31 (B) vs. 2.08 ± 1.31 (C); *p* = 0.0014). Moreover, among Polish participants, significant differences between bariatric or endoscopic and conservative treatment were noted in the context of loss of income (2.23 ± 1.12 (B) vs. 1.91 ± 1.11 (C); *p* = 0.0410) (Table 2). 

### 3.4. Associations between the Level of Perceived Discrimination and Sociodemographic and Clinical Factors 

The multiple regression analysis revealed that the level of discrimination due to obesity in both groups was associated with sex, age, degree of obesity, and treatment-related weight loss (*p* < 0.05) (Table 3). On the other hand, no associations were revealed with the level of education, duration of obesity, or the type of performed bariatric surgery or endoscopic method. Female sex compared with male sex was associated with a significantly higher level of discrimination. The level of discrimination was inversely correlated with age and weight loss, while a positive correlation was noted for body mass index (Table 3). 

## 4. Discussion

This study presents the current view on the level of perceived discrimination due to morbid obesity in two European countries. Such a comparison provides not only insights into the problem of obesity stigma, but also the basis for future research into the level of perceived discrimination in other countries. The valuable part of the study was the assessment of the impact of morbid obesity on the social and professional life. Good relationships, appropriate income, and social status are certainly factors that determine the quality of life in the population of obese individuals. When looking at the standardized questionnaires available for assessing the level of discrimination, it becomes clear that most of them are applicable to the general public. The questionnaire used in the presented study is certainly specific and limited to the key barriers that people with morbid obesity may face in everyday life.

The presented study showed no significant differences in the level of perceived discrimination due to morbid obesity between German and Polish participants. The lack of data for other European countries makes it impossible to compare our results with those from other studies. These gaps warrant further research in this area. In a study by Pearl et al., over 50% of participants reported at least one form of recurrent discrimination, 30% reported two or more reasons for discrimination, and 28.7% reported weight discrimination. Of the participants who experienced weigh discrimination, over 80% reported also at least one other form of discrimination [26]. In a cross-sectional study, Cedillo et al. reported that African American women felt more discriminated due to obesity than European American women [27]. 

A disparaging depiction of obese people by the media (e.g., as unpopular and unattractive) as well as the negative perceptions among the public (e.g., about incompetency and laziness) seriously affect the way people with obesity function on the employment market. In our study, twice as many German patients admitted that obesity was the reason for employment problems (to a high or very high degree) as compared with Polish patients. As for the loss of income in relation to obesity, this was reported less often by German participants, but there was a significant difference concerning the average level of discrimination between the two countries. Our findings are in line with previous studies that documented associations between perceived weight-based discrimination and problems experienced by employees [28,29,30]. Roehling et al. reported that employees with a BMI over 30 kg/m^2^ are even 37-fold more likely to suffer from employment discrimination than their normal-weight counterparts. Moreover, they concluded that given the particularly high levels of weight bias and discrimination in an employment setting, antibullying legislation is needed to develop a novel stigma-prevention policy and improve the situation of overweight people in the workplace [28]. 

A few experimental studies showed that inequities and disparities resulting from weight discrimination persist even when normal-weight and overweight applicants have similar job qualifications and credentials and, in some cases, even when normal-weight applicants have worse qualifications [30,31,32]. Our findings concerning obese employees are also in line with previous research conducted by Cawley et al. and Maranto et al. [33,34]. Contrary to most studies reporting the loss of income due to obesity, Lee et al. observed that obese and overweight men in Korea were 1.46-fold more likely to have professional jobs and had 13.9% higher monthly wages compared with normal-weight employees [35].

Weight discrimination has been described as a common form of mistreatment in many aspects of social life. In our study, more patients from Germany than from Poland declared that obesity was associated with the feeling of social exclusion and discrimination to a large or very large extent. However, the mean level of discrimination related to the sense of social exclusion was similar in both groups. Wear et al. also indicated that obese people are a common target of prejudice and often have problems with establishing satisfying social relationships [36]. 

The important finding from our study is that most participants in both countries reported the feeling of shame to a great extent due to the lack of attractive appearance, and there was a similar mean level of discrimination associated with shame. Moreover, almost one-fifth of German participants and more than one-sixth of Polish participants reported that the occurrence of obesity was the reason for difficulties in establishing relationships to a very high and high degree, and the analysis revealed a similar mean level of discrimination related to this aspect. Correlations between body weight and selected aspects of personal relationships were examined by Boyes et al. in 57 dating or married couples [37]. The authors noted that overweight women had lower-quality relationships that were more likely to end. 

The available literature shows that weight-based stigmatization is associated with gender, age, BMI, body dissatisfaction as well as relationship and sexual dissatisfaction [37,38,39]. This is in line with our study, which showed an association between the level of discrimination due to obesity and gender, age, degree of obesity, and treatment-related weight loss. Finally, in an analysis of data from 33 studies on weight stigma and its consequences, Wu et al. showed a positive correlation between the level of discrimination and the degree of obesity, body image dissatisfaction, and stress parameters, and a negative correlation between weight stigma and the self-esteem of obese individuals [40]. 

In our study, female sex was a significant predictor of a higher level of weight discrimination. This finding is in line with the results of other studies [14,41,42]. Similarly, in a Taiwanese study that assessed the relationship between obesity and health-related quality of life, Huang et al. confirmed that overweight women had lower physical health-related quality of life than normal-weight women [43]. 

Our study revealed that the level of perceived discrimination was negatively correlated with age and weight loss, and positively with BMI. Similarly, Puhl et al. estimated that the prevalence of weight discrimination among adults with obesity ranged from 19% to 42%, with higher rates among individuals with higher BMIs [42]. On the other hand, a cross-sectional study on a young Spanish population aged 11–17 years revealed that an increase in BMI was associated with more psychological weight-related problems [44].

More research is needed to elucidate the association between the level of perceived discrimination and the type of treatment for morbid obesity. In our study, there was a significant difference in the level of discrimination between patients treated with bariatric surgery or endoscopic method and those treated conservatively in the context of employment problems. This is in line with findings reported by Karmali et al. who assessed data from 4864 patients after bariatric surgery [45]. They concluded that weight stigma in workplaces and everyday life experienced by patients is one of the numerous factors responsible for weight regain, the other being mental problems, physical inactivity, dietary noncompliance, and endocrinopathies, among others. Kinzl et al. studied 547 patients with morbid obesity who underwent bariatric surgery. The authors aimed to identify potential difficulties in coping with new demands after weight reduction. They showed that one of the potential mechanisms underlying these difficulties was the simultaneous presence of mental disorders such as depression, disordered eating pattern, or cluster C personality disorders [46]. As in our study, Hebl et al. reported a close association between the level of perceived discrimination and age, gender, and the degree of obesity [47]. Similarly to us, Annis et al. showed the association between the level of perceived discrimination and weight loss [16]. They reported that individuals who suffered from weight discrimination were 2.5-fold more likely to gain weight during follow-up. 

It is important to acknowledge the differences between the Polish and German healthcare systems. Obesity prevention programs in Germany are better financed than they are in Poland, which translates into a reduced scale of discrimination against German individuals with obesity. First, in 2015, the German government passed the Preventive Act (Präventionsgesetz) on prophylaxis as part of healthcare. It is estimated that the German health insurance and healthcare fund spend over EUR 500 million a year on health promotion and preventive healthcare. Moreover, the act increased the funding of support groups by approximately EUR 30,000,000 (European Agency 2019). Second, in Germany, the so-called Multimodales Therapiekonzept (the Concept of Multimodal Therapy; MMK) assumes that insurance companies cover the costs related to bariatric surgery only if the patient participates in multimodal treatment for at least six months (up to one year). The MMK encompasses nutritional, behavioral, and exercise therapy. One of the therapeutic opportunities for patients with obesity (BMI ≥ 40.00 kg/m^2^ or ≥35.00 kg/m^2^ in the presence of comorbidities) is the so-called “Obesity Balance”, which lasts six months and consists of various modules such as nutritional therapy (6 meetings), exercise therapy (12 visits), and psychoeducation (6 visits). The program is supervised by a doctor and involves interdisciplinary cooperation. It also includes psychoeducation aimed at changing patients’ behavior and teaching patients to cope with various psychological situations, for example, weight stigma [48]. 

No such programs are currently available to Polish patients, which may explain why German individuals with obesity report lower levels of perceived discrimination. Another possible explanation for the discrepancies is that German citizens are more likely to view obesity as a physical and mental disease. Hilbert et al. reported that agreement with the disease concept predicted a lower obesity stigma [49].

### Study Limitations

Our study has several limitations. First, although German and Polish participants completed the same survey, they may have interpreted the questions differently, for example, because of differences between the two countries in public health support for patients with obesity. Therefore, the self-reported attitudes of participants may vary and may not reflect the actual experience of weight stigma in everyday life. Second, we recruited mainly middle-aged and older participants, so the results cannot be extrapolated to younger age groups. Previous research has shown that the greatest stigma around excessive body mass is observed among younger individuals, and especially in this group, obesity discrimination has been linked to numerous consequences such as maladaptive health behaviors, distress, and other health issues [15,47]. Third, our study group included white individuals, so the findings may not apply to other ethnic groups. 

While we are aware that the above limitations warrant further research into perceived weight stigmatization in the EU, our study lays the basis for developing interventions that aim to protect people with morbid obesity against the harmful effects of weight discrimination. It is important that strategies for reducing obesity bias are also present in the healthcare sector [50].

## 5. Conclusions

Our comparison of German and Polish patients undergoing treatment for morbid obesity revealed no differences in the general level of perceived weight discrimination. Polish participants showed a higher average level of discrimination related to the loss of income. In both countries, over two-fifth of patients declared that the occurrence of obesity was associated with the feeling of social exclusion and discrimination to a large or a very large extent. The mean level of discrimination related to the employment problems and loss of income was higher among patients who underwent bariatric surgery or endoscopic method than among those treated conservatively, both in Poland and in Germany. Moreover, the level of perceived discrimination was associated with sex, age, degree of obesity, and treatment-related weight loss. 

In conclusion, our findings reveal that obesity may influence not only the physical status but also the mental, social, and economic well-being of patients. Individuals treated for morbid obesity often experience multiple forms of discrimination in their daily life. Therefore, there is a great need to reduce obesity stigma in society and alleviate the socioeconomic and psychological burden of obesity. Currently, considering that the COVID-19 pandemic has resulted in an increasing use of the Internet for work and social life, it is also important that public health policymakers pay attention to social media as a space that should be free from obesity-stigmatizing content [51]. Our findings offer some new insights that could inform policymakers in the EU when developing policies aimed at minimizing inequalities and disparities due to obesity stigma. 

## Figures and Tables

**Table 1:** Comparison of sociodemographic and clinical parameters between patients with obesity from Poland and Germany *n* = 564.

	Poland			Germany		
	Total	Conservatively Treated Patients	Bariatric or Endoscopic Treated Patients	Total	Conservatively Treated Patients	Bariatric or Endoscopic Treated Patients
**Group size *n* (%)**	354	269 (75.98)	85 (24.02)	210	138 (65.71)	72 (34.29)
**Sex:**						
**Male *n* (%)**	80 (22.60)	65 (24.16)	15 (17.65)	52 (24.76) (*p* = 0.5369) ^b^	30 (21.74)	22 (30.56)
**Female *n* (%)**	274 (77.40)	204 (75.84)	70 (82.35) *p* = 0.21049 ^a^	158 (75.24) (*p* = 0.7563) ^b^	108 (78.26)	50 (69.44) *p* = 0.16002 ^a^
**Age (years)**	45.20 ± 15.69	46.61 ± 16.09	40.77 ± 13.52 *p* = 0.0856 ^a^	45.70 ± 9.70 (*p* = 0.8541) ^b^	36.52 ± 9.49	34.12 ± 9.97 *p* = 0.5231 ^a^
**Body mass index (kg/m^2^** **)**	36.92 ± 8.12	37.62 ± 7.75	34.67 ± 8.89 *p* = 0.3657 ^a^	36.87 ± 10.06 (*p* = 0.9452) ^b^	39.67 ± 10.08	37.46 ± 9.95 *p* = 0.4698 ^a^
**Obesity grade 1** **(BMI: 30–34.9 kg/m^2^)** ***n*** **(%)**	99 (27.96)	97 (36.19)	35 (41.67)	57 (27.14) (*p* = 0.8876) ^b^	48 (35.04)	31 (43.67)
**Obesity grade 2** **(BMI: 35–39.9 kg/m^2^)** ***n*** **(%)**	105 (29.66)	74 (27.61)	16 (19.05)	53 (25.24) (*p* = 0.0681) ^b^	26 (18.98)	15 (21.13)
**Obesity grade 3** **(BMI ≥ 40 kg/m^2^)** ***n* (%)**	150 (42.38)	98 (36.20)	34 (39.29) *p* = 0.08963 ^a^	100 (47.76) (*p* = 0.0741) ^b^	64 (45.99)	26 (35.21) *p* = 0.0986 ^a^
**Duration of the obesity (years)**	17.67 ± 11.61	20.02 ± 11.99	18.40 ± 10.11 *p* = 0.3641 ^a^	17.00 ± 10.51 (*p* = 0.7561) ^b^	17.64 ± 11.62	16.02 ± 7.89 *p* = 0.6952 ^a^
**Vocational education:**						
**Low level *n* (%)**	74 (20.90)	64 (23.79)	12 (14.29)	47 (22.23) (*p* = 0.7863) ^b^	28 (20.58)	18 (25.35)
**Average level *n* (%)**	151 (42.66)	120 (44.58)	40 (47.62)	86 (41.06) (*p* = 0.8213) ^b^	59 (42.65)	27 (38.03)
**High level *n* (%)**	129 (36.44)	85 (31.63)	33 (38.10) *p* = 0.05903 ^a^	77 (36.71) (*p* = 0.1245) ^b^	51 (36.76)	27 (36.62) *p* = 0.41128 ^a^
**Self-assessed financial status:**						
**Good *n* (%)**	208 (58.76)	146 (54.27)	62 (72.94)	72 (34.28) (*p* < 0.0001) ^b^	45 (32.61)	27 (37.50)
**Average *n* (%)**	119 (33.62)	99 (36.80)	20 (23.53)	91 (43.33) (*p* < 0.0001) ^b^	63 (45.65)	28 (38.89)
**Bad *n* (%)**	27 (7.63)	24 (8.93)	3 (3.53) *p* = 0.03560 ^a^	47 (22.38) (*p* < 0.0001) ^b^	30 (21.74)	17 (23.61) *p* = 0.32036 ^a^
**Comorbidities:**						
**Type 2 diabetes mellitus *n* (%)**	75 (21.06)	69 (25.65)	14 (16.47) *p* = 0.16057 ^a^	60 (28.57) (*p* < 0.0001) ^b^	46 (33.33)	20 (27.77) *p* = 0.60321 ^a^
**Hypertension *n* (%)**	160 (45.48)	159 (59.11)	33 (38.82) *p* = 0.00107a	99 (47.61) (*p* = 0.1253) ^b^	67 (48.55)	36 (50.00) *p* = 0.0869 ^a^
**Dyslipidemia *n* (%)**	63 (17.79)	109 (40.52)	22 (25.88) *p* = 0.01483 ^a^	40 (19.04) (*p* = 0.1478) ^b^	24 (17.39)	15 (20.83) *p* = 0.0352 ^a^
**Hyperuricemia *n* (%)**	14 (3.95)	21 (7.81)	2 (2.35) *p* = 0.07535 ^a^	12 (5.71) (*p* = 0.0635) ^b^	60 (4.34)	4 (5.55) *p* = 0.5698 ^a^
**Coronary heart disease *n* (%)**	88 (24.85)	37 (13.75)	7 (8.24) *p* = 0.17878 ^a^	52 (24.76) (*p* = 0.8652) ^b^	38 (27.53)	16 (22.22) *p* = 0.07891 ^a^

Data are presented as mean ± standard deviation unless otherwise indicated. NS—no significance. Significance for *p* < 0.05. ^a^ (*p* value not in brackets)—a comparison between procedures: conservative versus surgical treatment. ^b^ (*p* value in brackets)—a comparison between countries: Germany versus Poland.

**Table 2:** A comparative analysis of the level of discrimination among obese patients from Poland and Germany depending on the type of treatment (*n* = 564).

Level of Discrimination	Poland (*n* = 354)				Germany (*n* = 210)			
	General	Conservative Treatment (C) *n* = 272	Bariatric or Endoscopic Treated Patients (B) *n* = 82	*p* Value	General	Conservative Treatment (C) *n* = 137	Bariatric or Endoscopic Treated Patients (B) *n* = 73	*p* Value
**General level of discrimination** **Scale (5–25)**	13.50 ± 3.78, 13	13.32 ± 3.75, 13	14.50 ± 3.84, 14	0.0562 *	13.08 ± 4.66, 13 *p* = 0.4282 ^#^	12.64 ± 4.53, 13	14.19 ± 4.88, 14.50	0.0660 *
**Employment** **problems** **Scale (1–5)**	2.01 ± 1.16, 2	1.93 ± 1.15, 1	2.43 ± 1.15, 2	0.0028	2.29 ± 1.35, 2 *p* = 0.0703	2.08 ± 1.31, 1	2.85 ± 1.31, 3	0.0014
**Loss of** **income** **Scale (1–5)**	1.96 ± 1.11, 2	1.91 ± 1.11, 2	2.23 ± 1.12, 2	0.0410	1.59 ± 1.00, 1 *p* = 0.0018	1.49 ± 0.88, 1	1.85 ± 1.24, 1	0.0904
**Sense of** **social exclusion and discrimination** **Scale (1–5)**	3.08 ± 1.33, 3.00	3.09 ± 1.34, 3	2.98 ± 1.27, 3	0.5046	3.10 ± 1.36, 3.00 *p* = 0.7917	3.02 ± 1.38, 3	3.32 ± 1.27, 4	0.2388
**Sense of shame** **Scale (1–5)**	4.08 ± 1.12, 4.00	4.04 ± 1.11, 4	4.25 ± 1.12, 5	0.1153	4.03 ± 1.15, 4.00 *p* = 0.7672	3.95 ± 1.23, 4	4.27 ± 0.87, 5	0.2965
**Problems in establishing relationships** **Scale (1–5)**	2.51 ± 1.16, 2	2.49 ± 1.15, 3	2.61 ± 1.19, 2	0.6171	2.34 ± 1.23, 2.00 *p* = 0.0718	2.38 ± 1.20, 2	2.24 ± 1.32, 2	0.3647

Data are presented as mean ± standard deviation (SD), median (ME). *—U Mann–Whitney test: conservative treatment versus bariatric surgery in Germany and in Poland, ^#^—U Mann–Whitney test: level of perceived discrimination in Poland vs. in Germany.

**Table 3:** Multiple regression analysis for the level of patient’s discrimination due to prevalence of obesity in Poland and Germany (*n* = 564).

Variable	Coeff.	95% CI	*p* Value
**Sex**			
Female	(ref)		
Male	−3.45	−6.71, −0.19	0.038
**Age**	−0.10	−0.19, −0.01	0.029
**Education**			
Primary	(ref)		
Vocational	−1.94	−7.07, 3.18	0.451
Secondary	1.08	−4.39, 6.56	0.694
Higher	−1.27	−6.57, 4.03	0.634
**Duration of obesity**	0.014	−0.098, 0.127	0.795
**Weight loss**	−3.39	−6.86, −0.08	0.045
**Type of performed bariatric surgery or endoscopic method**			
Gastric balloon (endoscopic method)	(ref)		
Laparoscopic adjustable gastric banding	−4.35	−13.84, 5.14	0.363
Laparoscopic Roux-en-Y gastric bypass	−8.19	−16.35, −0.03	0.059
Laparoscopic sleeve gastrectomy	−7.06	−15.26, 1.14	0.090
**BMI classification**			
Normal-weight	(ref)		
Overweight	−0.35	−3.86, 3.15	0.839
Obesity grade 1	1.68	−1.63, 5.00	0.314
Obesity grade 2	3.17	0.89, 5.45	0.007
Obesity grade 3	4.09	1.97, 6.21	0.000

ref- reference category; The coefficients for the given categories are calculated in relation to the reference category. The value of the estimated coefficients indicate how much more or less the discrimination value is in relation to the reference category, e.g., the discrimination level for men is 3.45 less than the discrimination level for women.

## Data Availability

Data are available from the corresponding author on reasonable request.
